# KYNA Derivatives with Modified Skeleton; Hydroxyquinolines with Potential Neuroprotective Effect †

**DOI:** 10.3390/ijms222111935

**Published:** 2021-11-03

**Authors:** Bálint Lőrinczi, István Szatmári

**Affiliations:** 1 Institute of Pharmaceutical Chemistry, University of Szeged, Eötvös u. 6, H-6720 Szeged, Hungary; lorinczi.balint@szte.hu; 2 Institute of Pharmaceutical Chemistry, Interdisciplinary Excellence Center, University of Szeged, H-6720 Szeged, Hungary; 3 MTA-SZTE Stereochemistry Research Group, Hungarian Academy of Sciences, Eötvös u. 6, H-6720 Szeged, Hungary

**Keywords:** kynurenic acid, modified hydroxyquinolines, Conrad–Limpach reaction, neuroprotection, modified Mannich reaction

## Abstract

Kynurenic acid (KYNA) is an endogenous neuroprotective agent of increasing importance. Several derivatives have already been synthesized, bearing an abundance of functional groups attached to the main skeleton in different positions. Several of these compounds have already been tested in biological evaluations, with several of them targeting the same receptors and biological effects as KYNA. However, these modified compounds build upon the unmodified KYNA skeleton leaving a possible route for the synthesis of new, potentially neuroprotective derivatives with heteroatom-containing ring systems. The aim of this review is to summarize the syntheses of KYNA derivatives with altered skeletons and to pinpoint an appealing transformation for future medicinal lead molecules.

## 1. Introduction

KYNA (kynurenic acid) is an endogenous product of the tryptophan (TRP) metabolism, a pathway known to be responsible for the production of nicotinamide adenine dinucleotide (NAD) and NAD phosphate [1,2]. In this pathway, TRP is converted into various compounds, including L-kynurenine, which can be metabolized in two separate ways. One furnishes KYNA, whereas the other gives 3-hydroxykynurenine and quinolinic acid, the precursors of NAD [3,4].

Among the important features of KYNA, it is one of few known endogenous excitatory amino acid receptor blockers with a broad spectrum of antagonistic properties in supraphysiological concentrations. It is well established that KYNA has a high affinity towards N-methyl-D-aspartate (NMDA) receptors. Moreover, it has recently been disclosed that KYNA shows an even higher affinity towards positive modulatory binding sites at α-amino-3-hydroxy-5-methyl-4-isoxazolepropionic acid (AMPA) receptor [5].

Since KYNA is a neuroprotective agent able to prevent neuronal loss following excitotoxic, ischemia-induced, and infectious neuronal injuries [6,7], there has recently been increasing interest in the synthesis and pharmacological studies of KYNA derivatives. The substitution of KYNA at positions 5–8 was achieved by starting from the corresponding aniline via the modified Conrad–Limpach method [8,9,10]. The hydroxy group at position four was transformed to ether [10,11,12] or amine functions [13,14,15], while the carboxylic function at position two was mostly modified into the corresponding esters [10,11,12] or amides [16,17,18,19,20,21].

**Figure 1:**
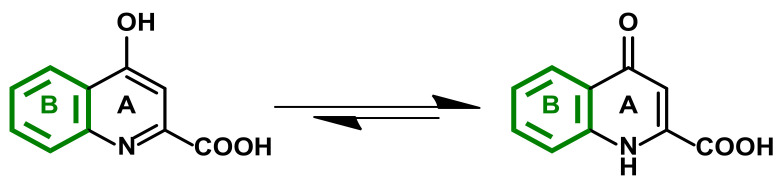
Structure of KYNA.

These modifications on the 4-hydroxyquinoline-2-carboxylic acid skeleton can either be (i) additions or modifications of functional groups at different positions or (ii) modifications in the benzo-fused 4-oxo-1,4-dihydropyridine-2-carboxylic acid skeleton itself. The latter can also be grouped into two distinct subgroups: the exchange of carbons to heteroatoms or the change of B ring size. While aspect (i) was already reviewed in 2009 [21], modifications on the B ring have not yet been collected. The aim of this review is to summarize the syntheses of KYNA derivatives modified at the B ring that either contain a heteroatom in the B ring and/or have a different ring size (Figure 1). In the future, these compounds may provide a basis for the synthesis of new KYNA derivatives and thus a variety of candidates for medicinal use.

With this in mind, the secondary aim of this review is to collect new substitution reactions carried out on the kynurenic acid skeleton. These new modifications may provide possibilities to improve pharmacological characteristics of the base compound, such as water solubility, blood-brain-barrier penetration and/or biological effects.

The synthesis of modified quinoline structures has a wide variety of possible methods [22]. However, with the 2-carboxylic and 4-oxo functions taken into consideration, the possible routes are narrowed down to a small range of nucleophilic substitutions or additions followed by intramolecular ring closure. Since the syntheses, from this aspect, differ only in a few cases, they have been categorized on the basis of the type and position of the heteroatom in the modified skeletons.

## 2. Synthesis of Functionalized and Heteroatom Containing KYNA Derivatives

### 2.1. Nitrogen-Containing Ring Systems

#### 2.1.1. Pyridine- and Pyrimidine-Fused Ring Systems

A possible route for the preparation of functionalized quinoline structures is the Conrad–Limpach synthesis. During the procedure described by Max Conrad and Leonhard Limpach, an aniline is reacted with β-keto esters to form an intermediate Schiff-base. In the second step, this intermediate undergoes a thermal intramolecular ring closure yielding the final quinolone derivative [23,24,25]. As the synthesis of the intermediate can be achieved with several methods, variations of the reaction using different aniline derivatives or electrophiles to synthesize the intermediate have been disclosed.

**Figure 2:**
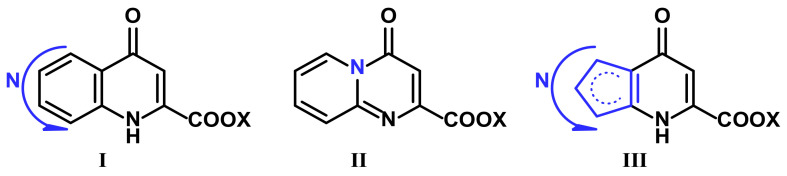
General formula for the Nitrogen-containing ring system.

**Scheme 1:**
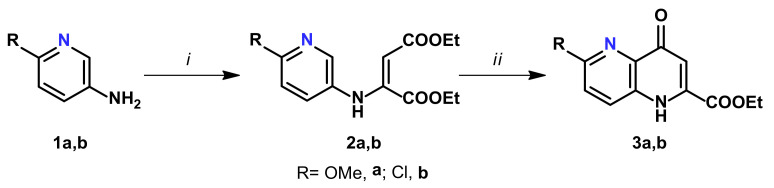
Synthesis of 1,5-naphthyridine derivatives **3a,b**.

One of the first reviews on the synthesis of KYNA derivatives bearing modified B rings (Figure 2. **I**) was published by Williamson et al. The reaction starting out from aniline derivative **1a** used ethyl ethoxalylacetate as β-keto ester, yielding intermediate **2a**. Then the product was stirred in boiling diphenyl ether (Ph_2_O) to achieve ring closure yielding 1,5-naphthyridine derivative **3a** (Scheme 1, Table 1) [26].

The same method was utilized 50 years later by Nakamoto et al. [27] and Feng et al. [28] in two distinct patents to synthesize the intermediates of **3b** by using either diethyl oxalacetate or its alkali salt, sodium (*Z*)-1,4-diethoxy-1,4-dioxobut-2-en-2-olate, respectively. The synthesized compounds were later transformed to potential antifungal [27] and antimalarial [29] or potential farnesoid X receptor modulators (Scheme 1 and Table 1) [28,30].

**Scheme 2:**
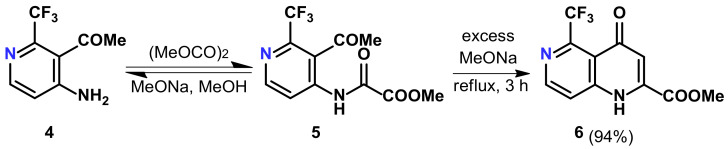
Synthesis of naphthyridine derivative **6** via acetophenone analog **4**.

In the case of the synthesis of 1,6-naphthyridine derivatives, the method presented in the literature [31] involves a procedure different from the one described by Conrad and Limpach. Pyridine with an active α-methyl function (**4**), which can be regarded as an acetophenone derivative [32], is reacted with dimethyl oxalate in the presence of excess MeONa needed to shift the reaction toward the ring closure (Scheme 2).

**Scheme 3:**
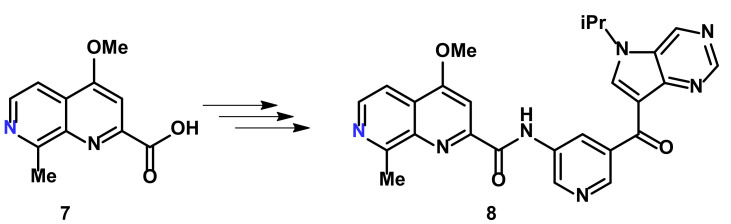
1,7-Naphthyridine derivative with possible kinase-inhibiting effect.

**Scheme 4:**
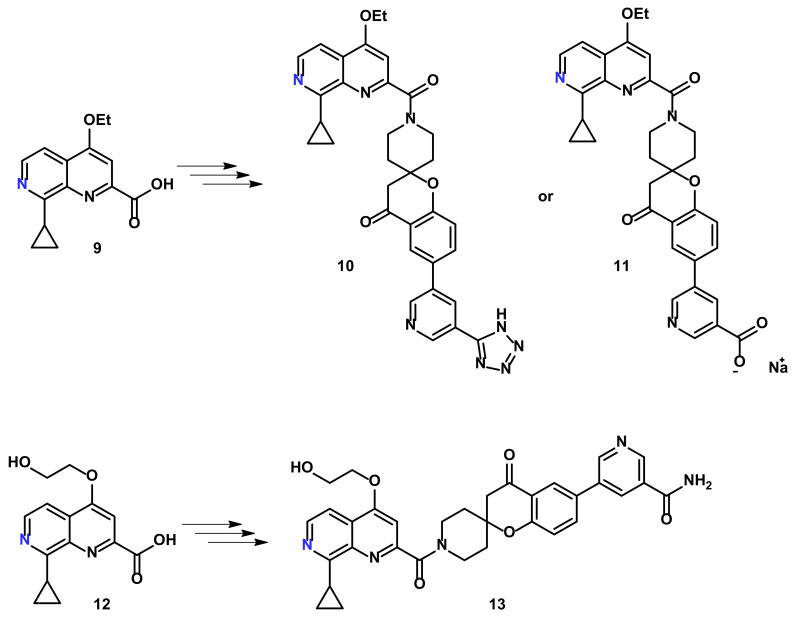
1,7-Naphthyridine derivatives (**10**, **11**, **13**) with possible acetyl coenzyme A inhibiting effect.

Regarding the synthesis of 1,7-naphthyridine derivatives, the literature is scarce: only two patents mention these compounds. In both cases, the derivatives were used to broaden the 4-hydroxyquinoline-2-carboxylic acid scaffold of the final bioactive compound with an additional heteroatom-containing skeleton (**8**, **10**, **11**, **13**, Scheme 3 and Scheme 4) [33,34]. However, the patents neither described the synthesis of the naphthyridine skeleton nor cited an appropriate reference.

**Scheme 5:**
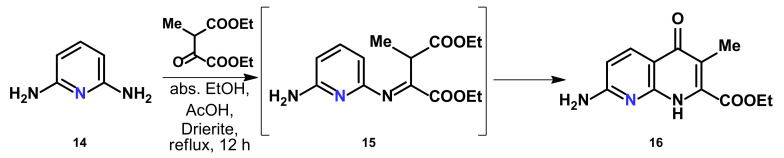
Synthesis of C-3 methyl-substituted 1,8-naphthyridine derivative **16**.

The synthesis of 1,8-naphthyridine, containing a carboxylic function at position C-2 and a hydroxy function at position C-4, was first carried out by Weiss et al. [35]. The reaction is a perfect example of the Conrad–Limpach procedure [23,24,25] using a substituted β-keto ester. For the formation of the Schiff base, α-ethoxalylpropionate (a β-keto ester) was applied, yielding **16**; however, its isolation in high purity was unsuccessful (Scheme 5).

**Scheme 6:**
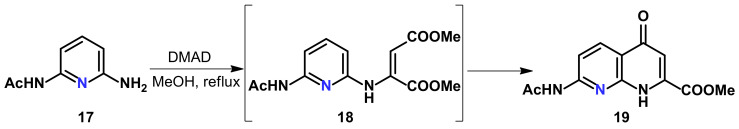
Proposed synthesis of 1,8-naphthyridine derivative **19**.

The modification used in the synthesis of many KYNA derivatives [36] was also applied for the synthesis of 1,8-naphthyridine derivatives. This method involves the use of acetylene derivatives for the synthesis of enamine intermediates through Michael addition. Tonetti et al. [37] utilized this method to synthesize **19** by applying dimethylacetylenedicarboxylate (DMAD) as an electrophilic reagent. However, the precise conditions of the synthesis are unclear, as the original paper could not be found, and the reaction scheme was put forward based on the information gathered from Chemical Abstracts (Scheme 6).

**Scheme 7:**
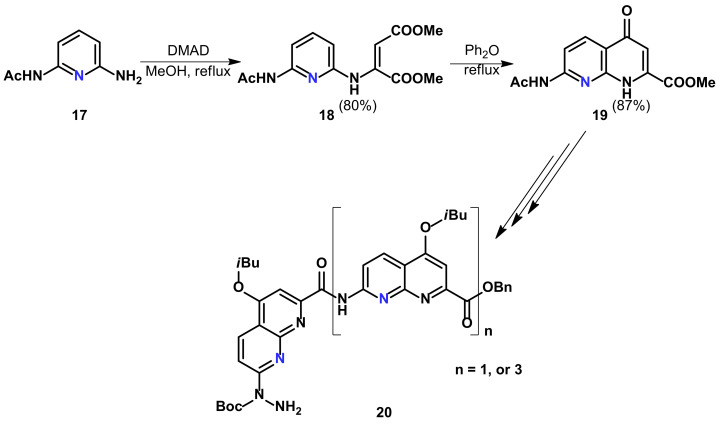
Synthesis of 1,8-naphthyridine derivative **19** and its use as a foldamer building block.

However, judging from the work of Huc et al., it is safe to conclude that the reaction depicted in Scheme 7 is correct, since the research group managed to synthesize the same compound from the same starting materials (Scheme 7) [38]. Derivative **19** was later used to create oligomers that were investigated in the hope of finding foldamers with a chemical space as vast as their aliphatic counterparts (namely, α-peptides) [39,40,41,42].

**Scheme 8:**
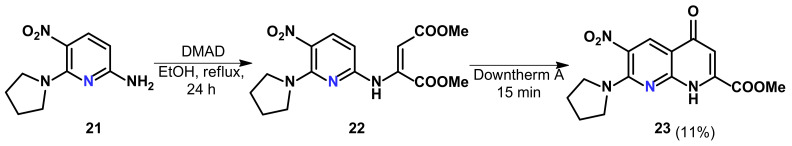
Synthesis of pyrrolidine substituted 1,8-naphthyridine **23**.

The same method was applied by Dohmori et al. [43] during the synthesis of different potentially antimicrobial agents (against *Trichomonas vaginalis*). Pyrrolidine-substituted 1,8-naphthyridine **23** was synthesized using functionalized aminopyridine **21** and DMAD, albeit with a low yield of 11%. Nevertheless, it further supports the idea that these compounds can be synthesized with this modified Conrad–Limpach procedure (Scheme 8).

**Scheme 9:**
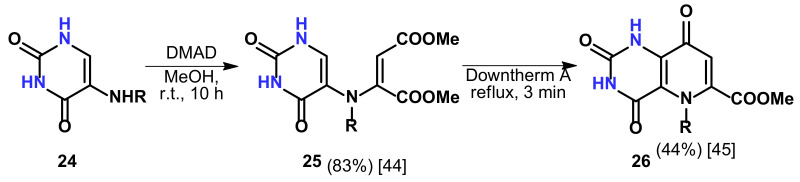
Synthesis of pyrido[3,2-*d*]pyrimidine derivative **26**.

The use of DMAD to carry out the Michael addition and, subsequently, the synthesis of different pyrimidine-containing skeletons have also been investigated. The first method published describes the synthesis of pyrido [3,2-*d*]pyrimidine skeleton [44]. This method was later optimized by Rosowsky et al. by decreasing the reaction time of the intramolecular ring closure from 45 min to a reaction time as short as 3 min (Scheme 9) [45]. The method was later cited by patents for the synthesis of compounds exhibiting PI3K inhibitory activities [46].

**Scheme 10:**
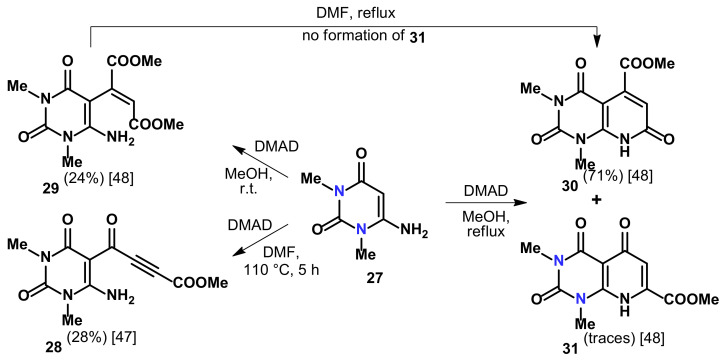
Possible methods for the synthesis of pyrido[2,3-*d*]pyrimidine derivative **31**.

The synthesis of the pyrido[2,3-*d*]pyrimidine skeleton has also been described; however, in the first publication, it was only mentioned as a desired product. The research group tried to apply the same Michael addition with DMAD. However, the reaction yielded the C-5 carbonylated derivative of 6-aminouracil (**28**, Scheme 10) [47]. Sakaguchi et al. tried to achieve the ring closure by changing *N,N*-dimethylformamide (DMF) to MeOH. Still, both at room temperature and under reflux conditions, the formation of the same maleate intermediate **29** was observed. Subsequently, it was transformed in either DMF or MeOH under reflux to 2-oxo-4-carboxylic acid derivative **30**. However, in MeOH, the formation of a minimal amount of the 4-oxo derivative **31** also took place. This observation, in combination with the fact that solvents used for other Conrad–Limpach ring closure steps [Dowtherm A, 1,2-dichlorobenzene (DCB)] were not investigated, indicates that the synthesis with a better yield may be possible (Scheme 10) [48].

**Scheme 11:**
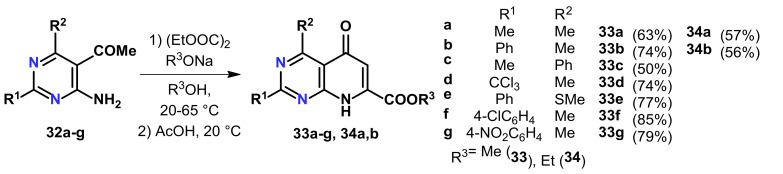
Synthesis of pyrido[2,3-*d*]pyrimidine derivatives **33a–g**, **34a,b** via acetophenone derivatives **32a–g**.

Dorokhov et al. used their method for 1,6-naphthyridine **6** [32] to synthesize a wide variety of pyrimidine analogues (**33a,b**, **34a–g**) as well (Scheme 11) [49].

#### 2.1.2. N-Bridgehead Annulations

For the synthesis of 4*H*-pyrido[1,2-*a*]-pyrimidin-4-one skeleton (Figure 2. **II**), several methods have been described. Earlier reports include the reaction of 2-aminpyridine with different reagents under varied conditions ranging from reactions at 250 °C using Meldrum’s acid [50] through reactions in AcOH (under reflux conditions) [51] or with the use of metal catalysts [52] to milder reactions in ethanol at reflux temperature [53]. However, the synthesis of quinoline compounds bearing both the same bridgehead nitrogen and the 4-oxo and 2-carboxylic functions required different conditions. In this subsection, the methods on the synthesis of the specific skeleton until now are collected.

**Scheme 12:**
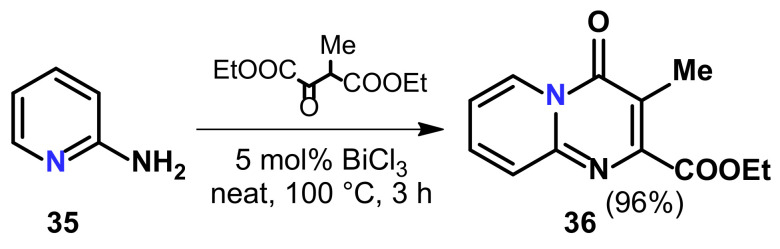
Synthesis of C-3 functionalized *N*-bridgehead derivative **36** using metal catalyst.

As a straightforward approach, the Conrad–Limpach procedure can also be applied to synthesize the required derivatives. Diethyl 2-methyl-3-oxosuccinate, as a functionalized β-keto ester, was used by Jaenicke et al. [54] along with bismuth trichloride as a catalyst to synthesize KYNA analog **36**, methyl-substituted at C-3 containing bridgehead nitrogen (Scheme 12). Regarding catalysts, the reaction showed high selectivity toward bismuth trichloride compared to other salts, such as zinc chloride or iridium chloride.

**Scheme 13:**
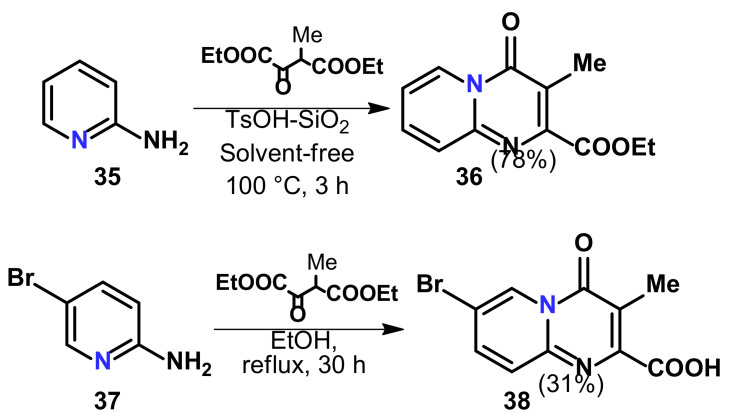
Metal-free synthesis of compound **36** and bromo-substituted derivative **38**.

It has been shown in two articles that the synthesis can also be carried out without the use of a toxic metal catalyst. Starting from the same β-keto ester, the synthesis of both **36** and the 6-bromo-substituted derivative **38** could be achieved (Scheme 13) [55,56]. However, the inaccurate description of the methods (no given *w*/*w*% and mol% of TsOH-SiO_2_ catalyst) and the lower yields overshadow the results.

**Scheme 14:**
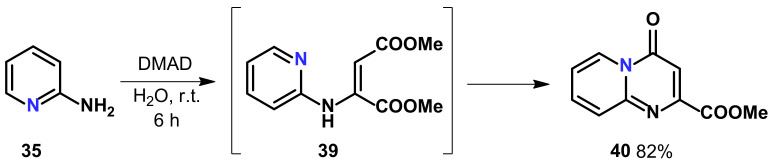
Synthesis of unsubstituted *N*-bridgehead derivative **40**.

Similar to the synthesis of pyridine-fused derivatives, the β-keto ester electrophile can be replaced by other electron-deficient reagents, such as DMAD. One of the reactions described used the acetylene derivative in water. The reaction carried out at room temperature yielded the unsubstituted *N*-bridgehead derivative **40** in 6 h (Scheme 14) [57]. This procedure was later applied in a patent to create derivatives inhibiting MeTTL3 activity, and in this way, also inhibiting proliferative disorders, such as cancer, autoimmune, infectious or inflammatory diseases [58].

**Scheme 15:**
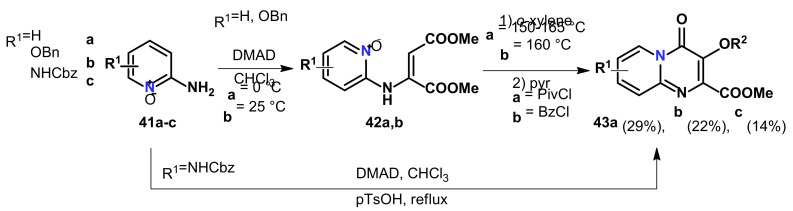
Synthesis of C-3 aryl- and alkoxy-substituted derivatives **43a–c**.

A similar procedure was used by Summa et al. [59,60] to synthesize 3-hydroxy-containing 9a*H*-pyrido[1,2-*a*]pyrimidine derivatives (Scheme 15). Based on the Conrad–Limpach procedures using DMAD as described in previous paragraphs, it is expected that the first step would involve the enamine formation of the acetylene and the amino group. However, based on their previous experiences with the synthesis of hydroxypyrimidinones, the formation of an *O*-adduct intermediate was expected. This could possibly be a 2-aminopyridine-*N*-oxide regarded as the tautomer of amidoxime. These derivatives are known to react with DMAD to form *O*-adducts (vinylhydroxylamines) [61,62,63,64,65]. The subsequent rearrangement/cyclization of **42a,b** took place in *o*-xylene under reflux. However, the isolation of the product was not described since it was further transformed without isolation into its pivalate derivative (**43a**) in order to facilitate its purification. The procedure was successfully broadened to other aminopyridine derivatives as well, with *N*-oxidation of the aminopyridines. It is interesting to mention that in the case of **43c**, the described final synthesis using *p*-toluenesulfonic acid (*p*TsOH) as a catalyst was performed in a single step, providing the lowest yield.

**Scheme 16:**
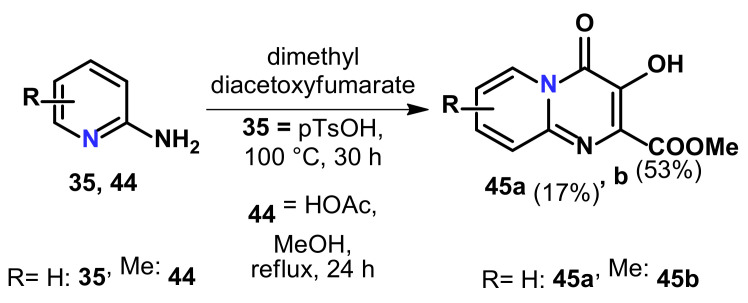
Synthesis of C-3 hydroxy-substituted *N*-bridgehead derivatives **45a,b**.

Furthermore, in addition to DMAD, the synthesis of the same C-3 hydroxy-functionalized compounds can be achieved through the use of a different electrophile, namely, dimethyl diacetoxyfumarate. Upon reacting with substituted 2-aminopyridines, it yields 2-hydroxy-pyrido[1,2-*a*]pyrimidin-4-on-2-carboxylic acid derivatives (**45a,b**, Scheme 16) [66]. The method was later applied for the synthesis of different HIV-1 integrase inhibitors [67,68,69].

**Scheme 17:**
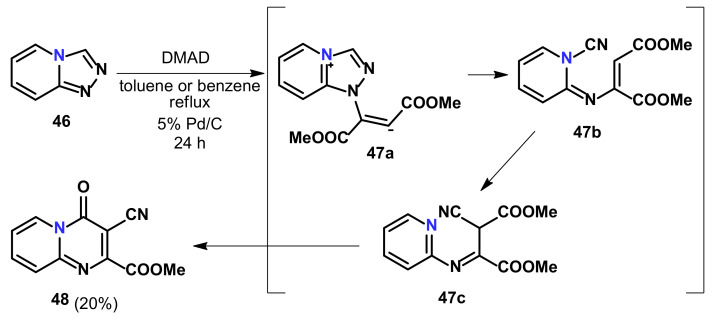
Synthesis of *N*-bridgehead KYNA derivative **48** via ring-member extension.

In continuing the discussion of reactions utilizing acetylenes to yield the desired pyrido[1,2-*a*]pyrimidin skeleton, DMAD can also be reacted with nucleophiles derived from unique compounds. An interesting alternative method was investigated by Rees et al. using 1,2,4-triazolo[4,3-*a*]pyridine as the nucleophile (Scheme 17) [70]. Among other reaction routes yielding different products, DMAD can react with the *N*-1 atom of the triazole ring, yielding intermediate **47a** delivering **48** through a ring-opening and subsequent intramolecular ring-closure.

**Scheme 18:**
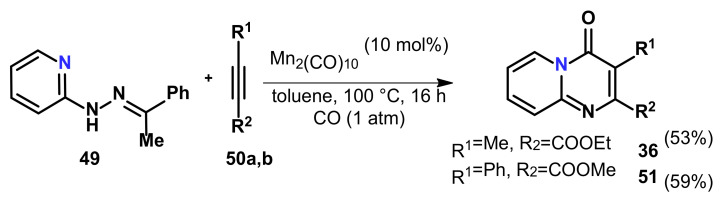
Synthesis of derivatives **36** and **51** via a manganese-catalyzed reaction.

Even though previous methods described above give possibilities for the synthesis of C-3-substituted derivatives, further functionalization can be achieved through the method reported by Ackermann et al. [71]. Starting from 2-pyridylhydrazone **49** and alkyne **50a,b**, manganese-catalyzed carbonylative annulations yielding compounds **36** and **51** were carried out (Scheme 18). The method was mainly used for the synthesis of non-carboxylic derivatives; however, judging from the number of available acetylene derivatives, this reaction may provide a useful approach to synthesize new, C-3 functionalized *N*-bridgehead KYNA derivatives.

**Scheme 19:**
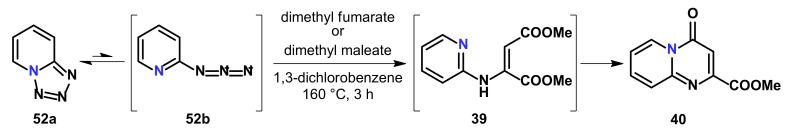
Synthesis of **40** from an azide starting compound.

One of the first publications regarding the synthesis of the pyrido[1,2-*a*]-pyrimidine skeleton was published in 1969 by Sturm et al. [72]. An azide derivative, a special nucleophile, was used as starting compound upon reacting with either dimethyl fumarate (64% product yield) or dimethyl maleate (59% yield). The following steps were proposed for a possible mechanism. First, tautomer **52b** reacts with one of the electrophiles through a Diels–Alder type forming a 5-membered ring. In subsequent ring-opening and elimination of N_2_, enamine intermediate **39** is formed, which, after a thermally driven ring closure, gives **40** as the final product (Scheme 19). The reaction between aminopyridine and acetylenedicarboxylic acid esters resulting in the same enamine (**39**) and giving the same product (**40**) after cyclization at 140 °C was also mentioned. However, neither a synthesis description nor a literature reference were included.

**Scheme 20:**
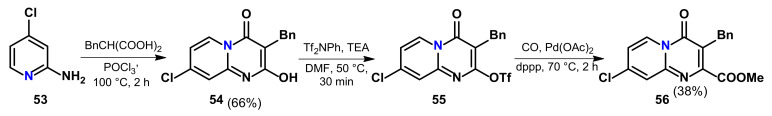
Further method for the synthesis of C-3 substituted *N*-bridgehead derivatives.

The use of malonic acid derivatives was also applied to synthesize derivatives bearing C-3 substitutions. However, this method requires further modifications since ring closure yields only 2-hydroxy derivative **54** that needs to be oxidized (Scheme 20) [73]. Even though the procedure itself is not as elegant as the previous ones, the use of functionalized anilines and malonic acids may yield a wide range of derivatives.

**Scheme 21:**
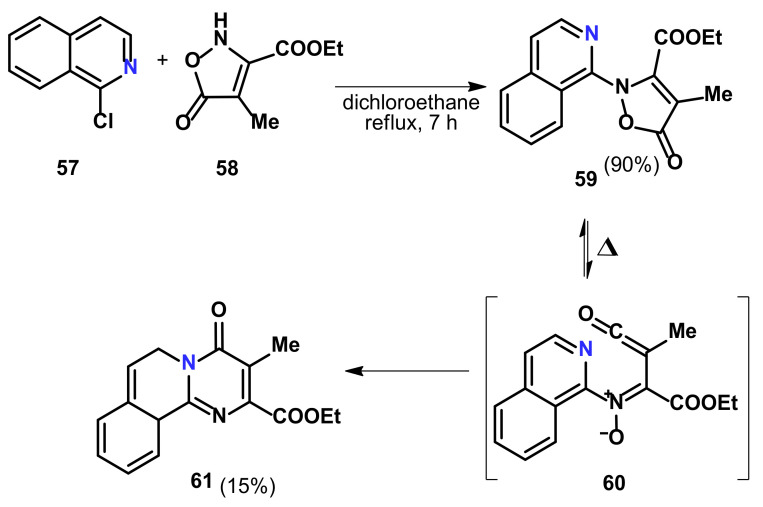
Pyrolytic decomposition of compound **59** yielding *N*-bridgehead derivative **61** of KYNA.

It is worth mentioning that the formation of the pyridine-fused pyrimidine skeleton has also been observed during the pyrolysis of an isoxazoline-substituted isoquinoline (**59**). The research group investigated the photolysis of different isoxazoline-substituted derivatives. When the photolysis of **59** was changed to pyrolysis in a scale-up study, the formation of **61** was observed (Scheme 21) [74].

**Scheme 22:**
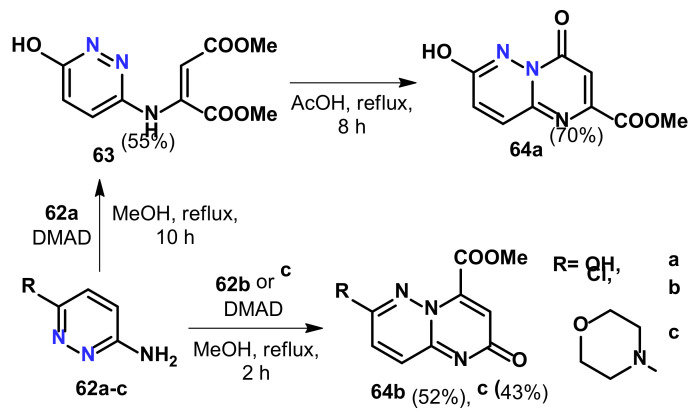
Synthesis of pyrimido[1,2-*b*]pyridazine derivative (**64a**) of KYNA.

Six-membered heterocycles containing more heteroatoms have reduced reactivity toward electrophiles. As a consequence, during the synthesis of KYNA skeletons with pyridazine, pyrimidine or pyrazine moieties, aniline starting materials may require further modifications. One such modified method applied by Mátyus et al. uses DMAD, and the accompanying aminopyridazine was substituted with electron-donating groups (EDG). However, besides electron donation, further factors may be present, as ring closure led to the formation of the desired pyrimido[1,2-*b*]pyridazin-2-one derivative only in the case of the hydroxy-substituted starting compound (**64a**). The morpholino group yielded 2-oxo-4-carboxylate analogues, similar to the chloro-substituted derivative (**64b,c**, Scheme 22) [75,76]. The research group also noted this difference as an anomaly, based on previous work [77]. These pieces of information and results with electron-density calculations support the conclusion that the favored reaction would be a nucleophilic addition on the C-3 of DMAD.

**Scheme 23:**
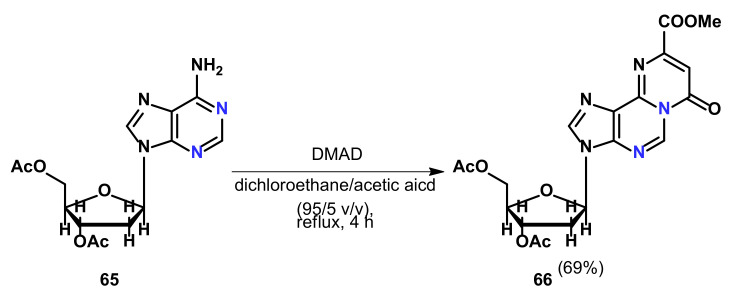
Synthesis of adenosine derivative **66**.

Koomen et al. [78] also used DMAD to synthesize adenosine derivatives bearing fluorescent attributes (Scheme 23). The starting 2′-deoxyadenosine compound **65** can be considered to be an imidazole-fused 6-aminopyrimidine. As previously mentioned, pyrimidine derivatives should be less reactive and thus unwilling to undergo the Michael addition; however, the imidazole ring may induce an excess of electrons of the pyrimidine ring, thus promoting the reaction.

**Scheme 24:**
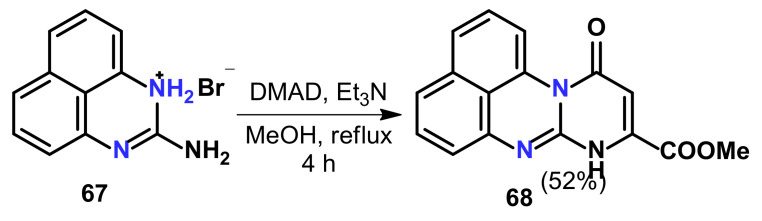
Synthesis of perimidine derivative **68**.

The work of Liu et al. [79] also seems to support the hypothesis that an electron-donating substituent favors the Michael addition. The synthesis of a special tetracyclic quinoline derivative [4(1*H*)-oxopyrimido[1,2-*a*]perimidine-2-carboxylate, **68**] was carried out starting from 2-aminoperimidine; hence, that can be considered to be 2-aminopyrimidine fused with naphthalene. Compared to the imidazole ring discussed previously, the naphthalene ring has a more prominent electron-donating quality. Consequently, the reaction could be carried out in MeOH (Scheme 24).

**Scheme 25:**
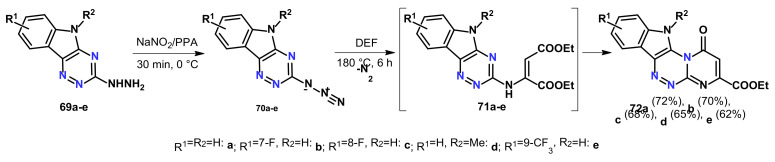
Synthesis of indole-fused *N*-bridgehead derivative **72a–e** from azide starting compounds.

The work of Jain et al. can be regarded as a further development of the study by Sturm et al. described in Scheme 19, except that it was implemented in the synthesis of bridgehead derivatives [80]. Through a different procedure, an azide intermediate was generated in both studies and was then reacted further with a fumaric acid ester (in this case, diethyl fumarate, DEF). Besides the hypothesized steps described by Sturm et al., an alternative route has also been proposed. First, the decomposition of the azide function takes place, and then a subsequent DEF attack provides an aziridine ring that opens up to give the enamine intermediates **71a–e** (Scheme 25).

#### 2.1.3. Five-Membered B-Ring Derivatives

**Scheme 26:**
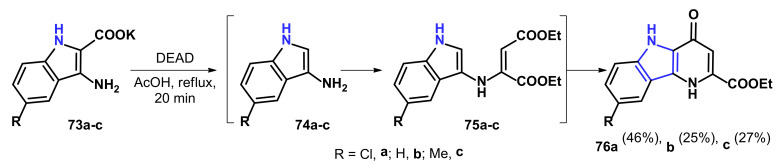
Synthesis of δ-carboline KYNA derivatives **76a–c**.

**Scheme 27:**
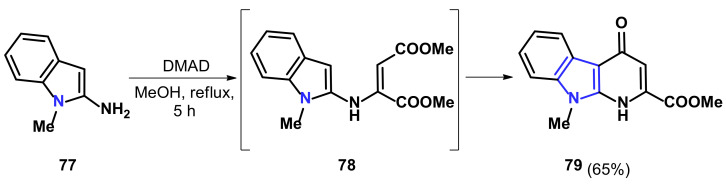
Synthesis of α-carboline KYNA derivative **79**.

Since five-membered heterocyclic compounds have an excess of electrons, the modified Conrad–Limpach procedure applying DMAD or DEAD (diethyl acetylenedicarboxylate) for the intermediate formation and subsequent ring closure can be easily carried out. Both pyrrolo[2,3-*b*]pyridine and pyrrolo[3,2-*b*]pyridine ring systems (Figure 2. **III**) have been synthesized in a similar way. Grinev et al. used the potassium salt of 3-aminoindole-2-carboxylic acid in glacial acetic acid yielding 3-aminoindole in situ that subsequently reacted with DEAD (Scheme 26) [81]. The reaction yields δ-carbolines **76a–c,** similar to the reactions described by Guyot et al. reporting the synthesis of α-carboline derivatives **79** (Scheme 27) [82].

It is interesting to note that in both cases, likely as a consequence of the high nucleophilicity of indole, the formation of side-products was also reported. In the synthesis of δ-carbolines, pentacyclic aromatic compounds appeared bearing an additional indole structure. In the case of α-carboline derivatives, in turn, Guyot et al. used *N*-methyl-2-aminoindole to presumably inhibit the formation of bridge-head nitrogen-containing derivatives. It was not directly specified by Guyot et al. whether it was for this reason. Nevertheless, in the following paragraphs, they described the synthesis of derivatives, where the ring closure takes place at the unsubstituted N-1 atom.

**Scheme 28:**
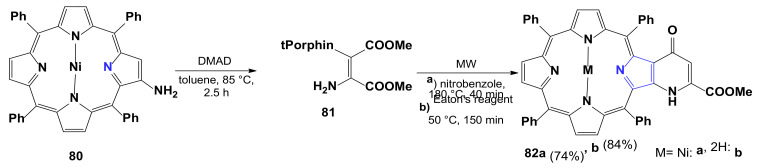
Synthesis of tetraphenylporphin derivatives **82a,b**.

The same Conrad–Limpach procedure can also be used for the synthesis of the pyrrolo[3,4-*b*]pyridine skeleton. Silva et al. prepared a tetraphenylporphin (tPorphin) compound containing the pyridine-2-carboxylic acid moiety fused to one of the pyrrole rings of the porphin skeleton (**82a,b**, Scheme 28) [83].

### 2.2. Sulfur-Containing Ring Systems

**Figure 3:**
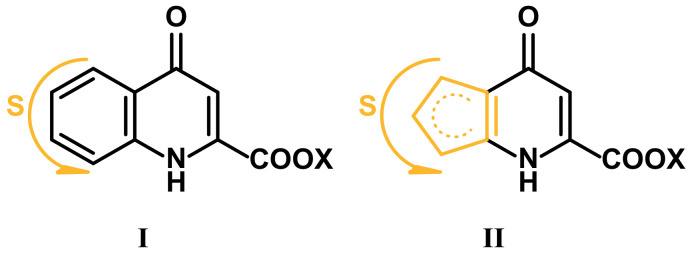
General formula for the Sulfur-containing ring system.

**Scheme 29:**
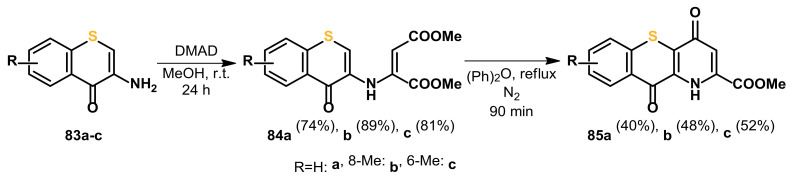
Synthesis of 10*H*-thiochromeno [3,2-*b*]pyridine derivatives **85a–c**.

Both six- and five-membered derivatives (Figure 3. **I** and **II**) have been synthesized. Kitao et al. used a modified Conrad–Limpach procedure, utilizing DMAD to form enamine intermediates (**84a–c**); ring formation was carried out in Ph_2_O (Scheme 29) [84].

**Scheme 30:**
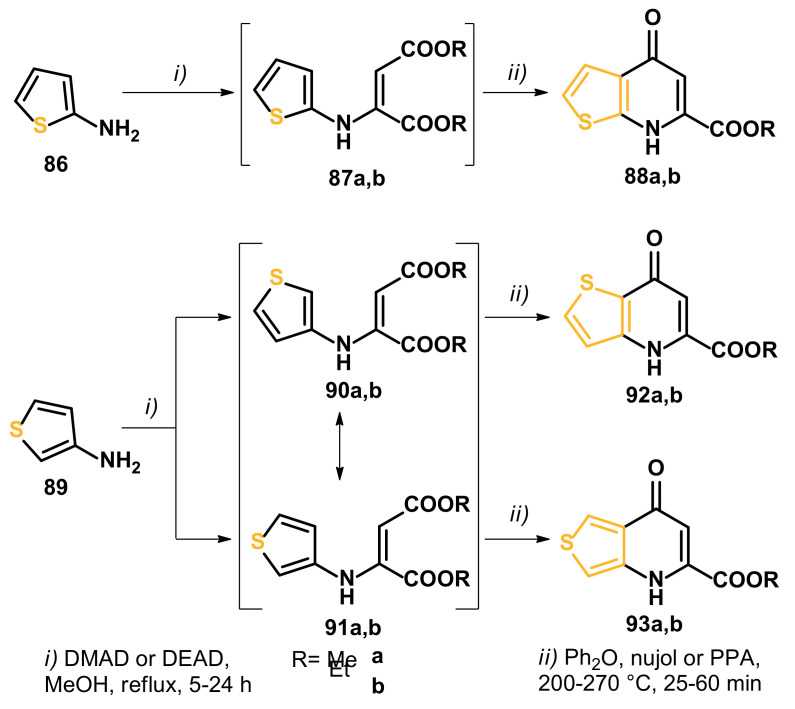
Synthesis of thiophene derivatives **88a,b**, **92a,b** and **93a,b**.

Baron et al. fabricated a wide variety of excitatory amino acid antagonists with the six-membered B ring of KYNA changed to a five-membered sulfur-containing ring. The synthesis of these compounds is similar to that of the six-membered skeleton, utilizing DMAD or DEAD in the first step with the corresponding aminothiophenes (**86,89**). The subsequent intramolecular ring closure was carried out in Ph_2_O, nujol or polyphosphoric acid (Scheme 30) [85]. Unfortunately, no yields have been published for compounds **88a,b**, **92a,b**, **93a,b**.

**Scheme 31:**
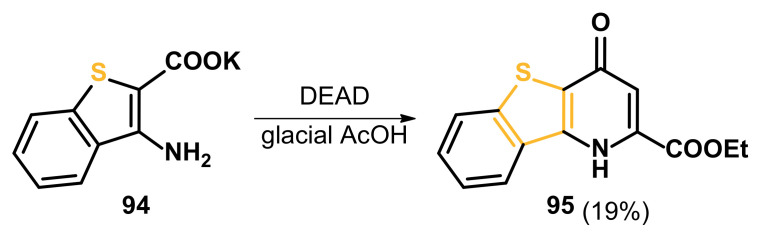
Synthesis of the thieno analog of δ-carboline derivative **76b**.

Although the method described in Scheme 30 has the most detailed description, it is not the only one. The synthesis of a thieno[3,2-*b*]pyridine system was already published earlier. Here, similar to the δ-carboline derivatives [78], DEAD was used, yielding the thieno analog (**95**) of δ-carboline derivative **76b** (Scheme 31) [86].

**Scheme 32:**
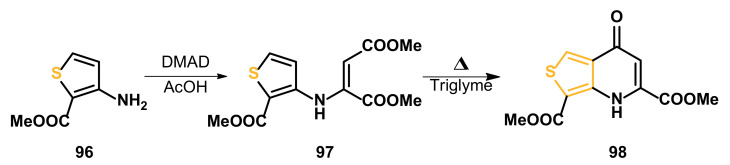
Synthesis of thieno[3,4-*b*]pyridine derivative **98**.

The thieno[3,4-*b*]pyridine skeleton was already mentioned earlier. However, in this work, the synthesis yield was not reported (Scheme 32) [87].

### 2.3. Oxygen-Containing Ring Systems

**Figure 4:**
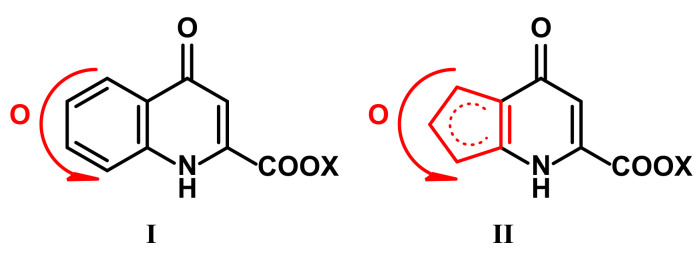
General formula for the Oxygen-containing ring system.

Regarding pyrano ring systems (Figure 4. **I** and **II**), it must be emphasized that, contrary to nitrogen-bearing systems, the aromaticity of the compound is lost. Whereas the procedures described in the following paragraphs indicate that the efficiency of the synthesis is not affected, the potential biological activity may vary substantially in comparison with KYNA.

**Scheme 33:**
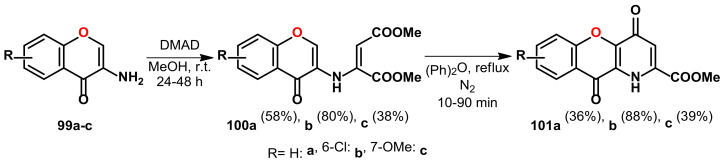
Synthesis of benzopyranopyridine derivatives **101a–c**.

Kitao et al. based their procedure for the thiopyrano derivatives [81] on the work of Strandtmann et al. [88], who synthesized 1,5-dihydro-4,10-dioxobenzopyranopyridine-2-carboxylic acid derivatives **101a–c** (Scheme 33).

**Scheme 34:**
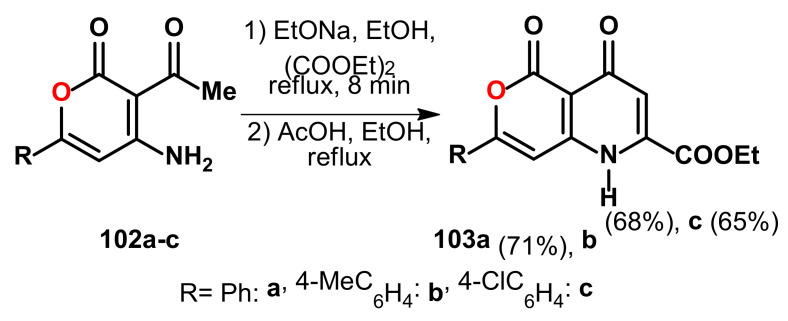
Synthesis of pyrano[4,3-*b*]pyridine compounds **103a–c** via acetophenone derivatives.

Dorokhov et al., besides synthesizing the pyridine- and pyrimidine-fused derivatives with the use of acetophenones [31,32], also achieved the synthesis of pyranone derivatives. Pyranones **102a–c** bearing the crucial active α-methyl function, similar to the ones used for pyridine- and pyrimidine-fused derivatives synthesized previously, were reacted with diethyl oxalate in the presence of sodium ethoxide (Scheme 34) [89].

**Scheme 35:**
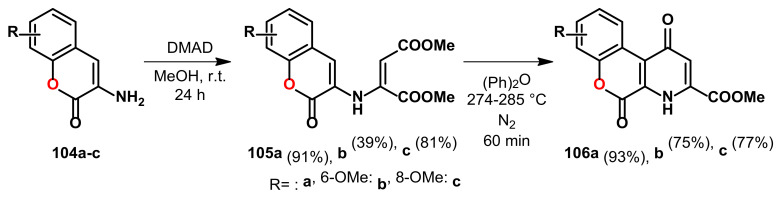
Synthesis of pyrano[3,4-*b*]pyridine derivatives **106a–c**.

The method used by Strandtmann et al. in the synthesis of benzopyrano[2,3-*b*]pyridine derivatives mentioned above was extended by the research group to synthesize benzopyrano[3,4-*b*]pyridine derivatives (**106a–c**, Scheme 35) [88]. The triazol derivatives of these compounds have been patented for their possible useful application in allergic manifestations such as bronchial asthma, hay fever, etc. [90].

**Scheme 36:**
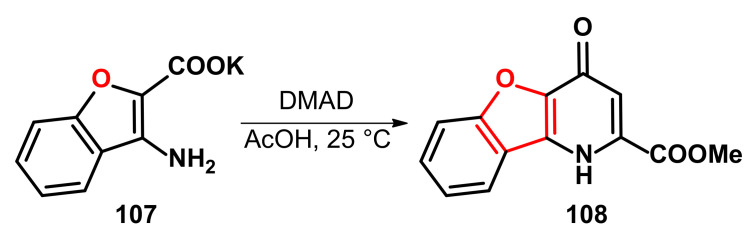
Synthesis of **108** the furan analog of δ-carboline **76b**.

Regarding the furan-fused 4-oxopyridine-2-carboxylic acid derivatives, the only synthesis mentioned describes a procedure similar to that applied in the synthesis of δ-carbolines reported earlier [82]. Using a potassium salt of 3-aminobenzofuran-2-carboxylic acid (**107**) and DMAD, compound **108**, the furan analog of δ-carboline **76b** could be synthesized (Scheme 36) [91].

### 2.4. New Aspects on the Synthesis of KYNA Derivatives

The aforementioned modified Conrad–Limpach procedure, applying DEAD and an aniline, has already been applied in the synthesis of KYNA [36] and several of its B-ring substituted derivatives [8,9,10].

**Scheme 37:**
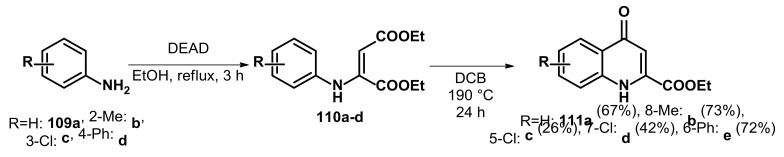
Synthesis of KYNA ethyl ester.

The synthesis applying this kind of C–L has recently been optimized by Szatmári et al. by applying two steps: (i) after the formation of enamine **110a**, column chromatography was used to purify the intermediate; (ii) Ph_2_O was replaced by DCB, which has a lower boiling point (180 °C) allowing an easier work-up procedure (Scheme 37) [92].

**Scheme 38:**
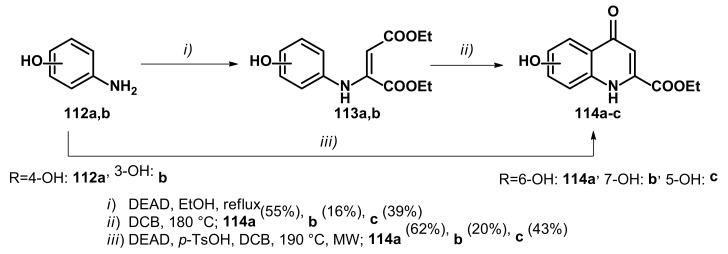
Synthesis of hydroxykynurenic acid derivatives.

The synthesis of the methyl ester analogs of **114a–c** has already been described [93,94]; however, no yields or exact synthetic procedures were detailed. Szatmári et al. successfully carried out the synthesis of compounds **114a–c**, applying the before mentioned optimizations, reaching higher yields [95]. Furthermore, based on the work of Sutherland et al. [96], *p*-TsOH as a catalyst was investigated in a one-pot version of the Conrad–Limpach procedure applying microwave irradiation (Scheme 38) [95]. The synthesis provided the appropriate hydroxy derivatives in increased yields with diminished maleimide formation, and the work-up could be carried out without time-consuming chromatography.

## 3. Mannich-Type Transformations of KYNA and Its Substituted Derivatives

### 3.1. C-3 Substitutions of KYNA Derivatives

**Scheme 39:**
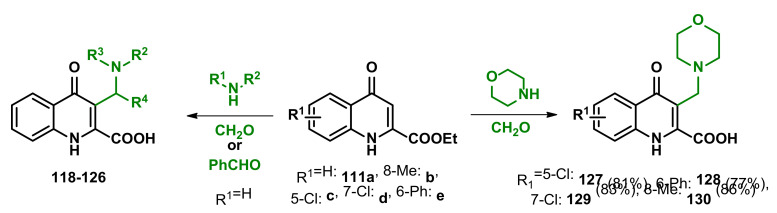
Synthesis of KYNA and B-ring substituted KYNA Mannich derivatives.

Formally, KYNA can be considered to be a nitrogen-containing 1-naphthol derivative. Based upon their previous works with 1-naphthol and its *N*-containing analogs [97,98,99,100], Szatmári et al. successfully applied the ethyl ester of KYNA in the modified Mannich reaction (*m*Mr) [101]. In this regard, to explore the scope and limitations of the reaction, different primary and secondary amines (acyclic and cyclic) were reacted with KYNA in the presence of formaldehyde (22% solution) in an optimized reaction condition of 1,4-dioxane as solvent under reflux conditions with a 5 h reaction time. The synthesis all yielded the C-3 aminoalkylated kynurenic acid derivatives **118-126** (Scheme 39, Table 2).

**Scheme 40:**
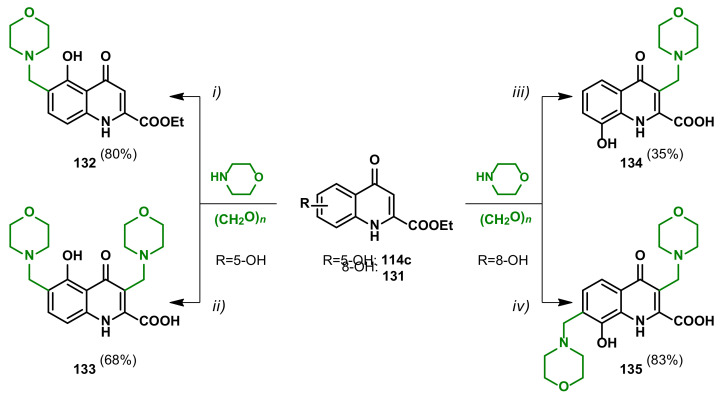
Aminoalkylations of the ethyl esters of 5- and 8-hydroxykynurenic acids. (**i**) **114c**:morpholine:(CH_2_O)_n_ 1:1:3; EtOH, MW, 150 °C, 1 h; *(**ii**)* **114c**:morpholine:(CH_2_O)_n_ 1:3:4.6; EtOH, MW, 80 °C, 2 h; (**iii**) **131**:morpholine:(CH_2_O)_n_ 1:1:3; dioxane, MW, 150 °C, 2 h; (**iv**) **131**:morpholine:(CH_2_O)_n_ 1:3:4.6; toluene, MW, 80 °C, 1 h.

To test the effect of substituents at positions 5, 6, 7 or 8 on the *m*Mr, derivatives **111b–e** were reacted with morpholine as a representative secondary cyclic amine in the presence of formaldehyde. Reactions were carried out under the optimized reaction conditions, concluding that aryl/alkyl substituents at position 6 or 8 and the halogen at position 5 or 7 have no significant influence on the aminoalkylation at C-3 (Scheme 40, **127–130**).

### 3.2. Aminoalkylations of Hydroxy-KYNA Derivatives

**Figure 5:**
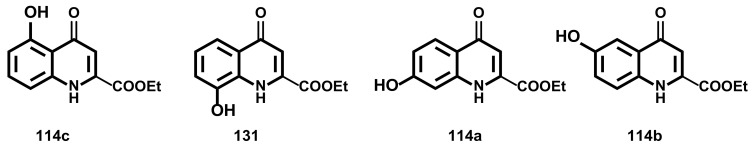
Structures of hydroxy-KYNA derivatives.

Regarding the 1-naphthol analogy of KYNA, the B-ring substituted derivatives show possibilities for further fascinating *m*Mr transformations. Compounds **114c**, **131** through their analogy with 1-naphthol and **114a,b** through their 2-naphthol analogy (Figure 5), offer new possible reaction routes for the aminoalkylation.

#### 3.2.1. Derivatives with Structural Similarities to 1-Napthol

Hydroxylated KYNA derivatives bearing the secondary hydroxy function at position 5 or 8 show a second 1-napthol similarity. In this case, the aminoalkylations can be expected to first undergo it in position 6 or 7, respectively, as these positions can both be regarded as the C-2 and, consequently, the most reactive position of a 1-napthol skeleton, as well as the sterically-less hindered position of the KYNA skeleton. However, this substitution route was observed by Szatmári et al. only in the case of compound **114c**, the reactions first yielding **132,** and subsequently **133**. The morpholinomethylation in the case of the 8-hydroxy derivative (**131**) first took place at C-3, yielding **134** and only after that at C-7 (Scheme 40, **135**) [95].

In both cases, DFT calculations were carried out to support the observed results, showing that the reason behind the sluggish and irregular reactivity of **131** might be due to its low HOMO energy level.

#### 3.2.2. Derivatives with Structural Similarities to 2-Napthol

**Scheme 41:**
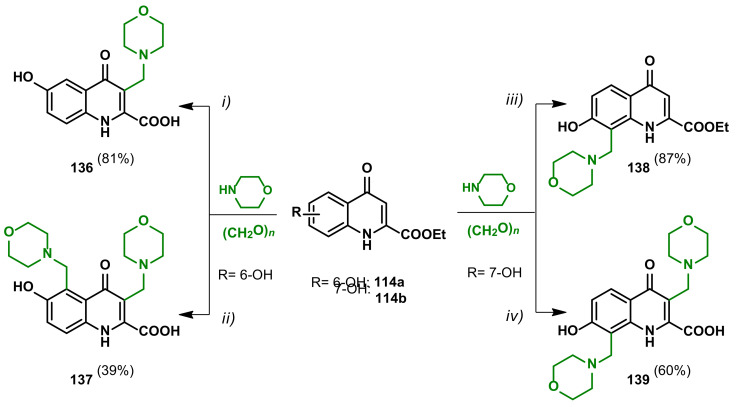
Morpholinomethylations of the ethyl esters of 6- and 7-hydroxykynurenic acids. (**i**) **114a**:morpholine:(CH_2_O)_n_ 1:3:4.6; EtOH, MW, 80 °C, 30 min; (**ii**) **114a**:morpholine:(CH_2_O)_n_ 1:3:4.6; EtOH, MW, 80 °C, 2 h; (**iii**) **114b**:morpholine:(CH_2_O)_n_ 1:1:3; EtOH, MW, 80 °C, 1.5 h; (**iv**) **114b**:morpholine:(CH_2_O)_n_ 1:2:4; EtOH, reflux, 56 h.

In the case of the 6- and 7-hydroxykynurenic acids (**114a,b**), a similarity between the skeleton and 2-naphthol can be observed, implicating that the aminoalkylations may take place in position 5 or 7, respectively. After performing the reactions, it has been observed that only compound **114b** shows the expected reactivity with the morpholinomethyl function first forming at position C-8 (**138**) and subsequently in position C-3 (Scheme 41, **139**). In the case of compound **114a,** the reaction first led to the formation of the C-3 substituted derivative and later to the formation of the disubstituted derivative (**136** and **137**). This unexpected reactivity was later investigated by DFT calculation disclosing HOMO delocalization and local NBO charges for the corresponding anion pairs theorized to form during the reaction. These calculations were in line with the experimental results implying that in the case of **114a,** despite the similarity to 2-naphthol, the additional functional groups and heteroatom in the ring-system may override the reactivity expected from the sterically-less hindered 2-naphthol skeleton, resulting in the formation of **136** as a primary product [95].

## 4. Conclusions

Kynurenic acid derivatives with a modified B-ring skeleton represent an interesting alternative for the synthesis of new KYNA analogs. Most of the compounds discussed have already been synthesized with the aim of biological use, such as antiviral, antimicrobial, antiallergic or antitumor effects; however, only a fraction of them have been tested for KYNA-like effects. The primary aim of this review was to summarize the procedures used for the synthesis of KYNA derivatives. These syntheses, combined with the second aim of the review—transformations, facilitating blood-brain-barrier penetration—may pave the way for future pharmaceutical chemistry concerning KYNA derivatives.

Summarizing the procedures used for the synthesis of B-ring modified KYNA derivatives, they build upon the reaction between a nucleophile (heteroatom-containing aniline/five-membered aromatic compound with an amine function) and an electrophile [β-keto ester/dicarboxylic alkyne (DEAD or DMAD)]. Based on the electrophiles, the reactions can be categorized as Conrad–Limpach (using β-keto esters) and modified Conrad–Limpach (using DEAD or DMAD) procedures.

While the unmodified procedure may give way for C-3 substitution of the KYNA skeleton without further transformations needed, the modified Conrad–Limpach procedure, compared to the prior, has the advantage of a cheaper electrophile and, combined with the recent optimization steps or one-pot reaction set-up may provide a more cost-efficient synthesis.

As KYNA is mainly known in the literature for its neuroprotective effect, the modified Mannich reaction, resulting in derivatives bearing one or more tertiary nitrogen and thus having better blood-brain-barrier penetration, rises as a possible further transformation. As KYNA and some of its derivatives can easily undergo this reaction, it is safe to hypothesize that the B-ring modified derivatives that can be fabricated with the procedures summarized in this review can also undergo the reaction and may provide potentially neuroprotective compounds.

## Figures and Tables

**Table 1:** Reaction conditions for the preparation of 1,5-naphthyridine derivatives.

Reaction	Reagent	Solvent	Temperature (°C)	Reaction Time	Yield (%)
*i*	diethyl oxalacetate	glacial acetic acid	40–50 °C; r.t.	4 h; overnight	**2a** 43% [26]
		neat	90 °C	7 h	**2b** 21% [27]
	sodium (*Z*)-1,4-diethoxy-1,4-dioxobut-2-en-2-olate	glacial acetic acid	r.t.	2.5 days	**2b** 23% [28]
*ii*	-	Ph_2_O	reflux	10 + 10 min	**3a** 84% [26]
		Dowtherm A	210 °C	5 h	**3b** 34% [27]
		Ph_2_O	reflux	15 + 20 min	**3b** 29% [28]

**Table 2:** KYNA Mannich derivatives.

Compound #	R^2^	R^3^	R^4^	Yield (%)
**118**	H	2-morpholinoethyl	H	78
**119**	H	2-(dimethylamino)ethyl	Ph	74
**120**	Me	Me	H	82
**121**	Me	Bz	H	85
**122**	morpholine		H	91
**123**	piperidine		H	73
**124**	*N*-methyl-piperazine		H	78
**125**	1,2,3,4-tetrahydroisoquinoline		H	81
**126**	6,7-dimethoxy-1,2,3,4-tetrahydroisoquinoline		H	72

## Abbreviations

Acetonitrile	MeCN
Blood-brain barrier	BBB
1,2-Dichlorobenzene	DCB
Diethylacetylene dicarboxylate	DEAD
Diethyl fumarate	DEF
N,N′-Diisopropylcarbodiimide	DIC
Dimethylacetylene dicarboxylate	DMAD
N,N-Dimethylformamide	DMF
Diphenyl ether	Ph_2_O
1,3-Bis(diphenylphosphino)propane	dppp
Electron-donating groups	EDG
1-Hydroxybenzotriazole hydrate	HOBt
Nicotinamide adenine dinucleotide	NAD
Modified Mannich reaction	mMr
Polyphosphoric acid	PPA
Tetraphenylporphin	tPorphin
TNF-stimulated gene 6 protein	TSG-6
p-Toluenesulfonic acid	pTsOH
Triethylamine	TEA
Tryptophan	TRP
Tumor necrosis factor alpha	TNF-α
